# *Candida Glabrata* Lymphadenitis Following Infliximab Therapy for Inflammatory Bowel Disease in a Patient With Chronic Granulomatous Disease: Case Report and Literature Review

**DOI:** 10.3389/fped.2021.707369

**Published:** 2021-10-25

**Authors:** Heather Kristin Lehman, Rahool Davé

**Affiliations:** University at Buffalo, Buffalo, NY, United States

**Keywords:** chronic granulomatous disease (CGD), infliximab, TNF - α, CGD colitis, *Candida glabrata*

## Abstract

Chronic granulomatous disease (CGD) is an inborn error of immunity caused by inactivating genetic mutations in any one of the components of the phagocyte nicotinamide adenine dinucleotide phosphate (NADPH) oxidase complex. Phagocytic cell reactive oxygen species generation is impaired in the absence of a functional NADPH oxidase complex. As a result, patients with CGD are at high risk of developing deep-seated infections with certain bacteria and fungi. Additionally, aberrant inflammation and granuloma formation may occur in multiple organs including the bowels, with inflammatory bowel disease seen as a common inflammatory complication of CGD. Traditionally, TNF-α inhibitors are considered effective biological therapies for moderate-to-severe inflammatory bowel disease. While limited case series and reports of patients with CGD have shown improvement in fistula healing with use of TNF-α inhibitors, several patients have developed severe, even fatal, infections with CGD-related pathogens while on TNF-inhibitor therapy. In this case report, we describe an adolescent male with X-linked CGD and steroid-refractory colitis with perirectal fistula and abscesses, who was initiated on treatment with infliximab, a TNF-α inhibitor. Following his first two infliximab doses, the patient developed a *Candida glabrata* lymphadenitis and associated ulcerating oropharyngeal lesions, requiring hospitalization and therapy with amphotericin B for resolution. We compare our patient's case to prior reports of infliximab use in CGD-related inflammatory bowel disease.

## Introduction

Chronic granulomatous disease (CGD) was first described in the 1950s in a series of four pediatric patients in Minnesota who were found to have a strikingly similar constellation of symptoms. These children presented with chronic suppurative lymphadenitis, hepatosplenomegaly, pulmonary granulomas, and eczematoid dermatitis (1). Since the initial disease description, CGD has been found to occur at a frequency of one out of every 200,000 live births in the USA (2). Genetic analysis has revealed CGD to be a diverse family of mutations in any one of the components of the phagocyte nicotinamide adenine dinucleotide phosphate (NADPH) oxidase complex. Phagocytes (neutrophils, macrophages, and monocytes) utilize the NADPH oxidase complex to generate reactive oxygen species (ROS) to destroy phagocytosed pathogens, primarily bacteria and fungi (3). The ROS generated by NADPH oxidase also mediate formation of NETs which trap extracellular microbes, and NET formation is abnormal in patients with CGD (4, 5). Additionally, neutrophils from CGD patients are reported to have decreased expression of toll-like receptors (TLR)5 and TLR9. This decreased TLR5 and TLR9 expression can be reproduced *via in vitro* inhibition of NADPH oxidase in neutrophils from health controls (6). These defects result in phagocytes which are unable to effectively kill intracellular and some extracellular microorganisms (7).

The NADPH complex is a five-protein complex. A transmembrane heterodimer of gp91^*phox*^ and p22^*phox*^ (encoded by the *CYBB* and *CYBA* genes, respectively, and known together as cytochrome b588) resides on the plasma membrane or the membrane of secondary and tertiary granules and acts as a scaffolding upon which the cytosolic complex of p47^*phox*^, p67^*phox*^, and p40^*phox*^ (encoded by *NCF1, NCF2*, and *NCF4* genes, respectively) organizes to form a functional NADPH complex. EROS is a protein that stabilizes the NADPH complex in the membrane of the endoplasmic reticulum and is encoded by the *CYBC1* gene. A defect in any one of these portions of the NADPH complex-associated proteins leads to the clinical phenotype of CGD (8, 9). Rac2 and Rap1 also aide in the function of the complex. Defective gp91^*phox*^ accounts for about 65% of cases of CGD in the USA. Mutations in gp91^*phox*^ are inherited in an X-linked manner, resulting in a male-predominance of CGD in American populations (10). Defects in the other proteins are inherited in autosomal recessive manner and are common in regions with higher rates of consanguinity (11–13).

Dysfunctional phagocytes in CGD cannot effectively destroy certain bacteria and fungi. Patients with CGD are highly susceptible to a relatively narrow spectrum of pathogens. The most common pathogens found in a North American cohort of patients with CGD were *Staphylococcus aureus, Serratia marcescens, Burkholderia cepacia, Nocardia* spp., and *Aspergillus* spp. (14). Infections in patients with CGD are often indolent due to the blunted innate immune response (6) and may be found in their more advanced stages (15).

In addition to bacterial and fungal infections, patients with CGD are at increased risk of granulomata formation in any hollow viscera. Granulomas are most often seen in pulmonary, gastrointestinal, and genitourinary systems in these patients (16). The immunological basis of granulomata formation is not well understood. Proposed mechanisms for hyperinflammation and granulomatous disease in CGD include impaired phosphatidylserine-dependent recognition and removal of apoptotic cells (efferocytosis) (17), and uncontrolled inflammasome activity (18, 19).

Therapies such as IFN-γ and antifungal/antibacterial prophylaxis have led to improved disease control and prolonged survival, with many patients with CGD living well into their young adult years and beyond (20, 21). Given this increase in life expectancy, additional immune dysregulatory sequelae of CGD are being observed, including an inflammatory bowel disease-like colitis (22). CGD-related colitis commonly features granuloma formation, mimicking Crohn disease, with other potential histologic features including pigmented macrophages, tissue eosinophilia, and neutrophilic inflammation (23). In a series of 140 patients with CGD, 32.8% of patients had IBD. X-linked CGD was the underlying defect in 89% of the subset of patients with CGD-related colitis, though only 67% of the total patient series had X-linked CGD (22).

TNF-α inhibitors are an important therapeutic drug class in non-CGD patients with moderate-to-severe IBD, and are beneficial in treating fistulizing disease (24). However, it is recognized that TNF-α inhibitors predispose patients without known immunodeficiency to infections with certain pathogens, such as *Mycobacterium* and dimorphic fungi. This increased risk is considered to be related to the role of TNF-α in granuloma formation and maintenance (25, 26). Several case reports and series have indicated infectious risks in patients with CGD treated with TNF-α inhibitors might be elevated and even life-threatening (27). We present the case of an adolescent male with X-linked CGD and steroid-refractory colitis with perirectal fistula and abscesses, who was initiated on treatment with infliximab, a TNF-α inhibitor. Following his first two infliximab doses, the patient developed a *Candida glabrata* lymphadenitis, and HSV-positive and *Candida*-positive ulcerating oropharyngeal lesions, requiring hospitalization and therapy with amphotericin B for resolution. Our patient's case highlights the risk of severe infections in patients with CGD treated with TNF-inhibitors, adding to the literature documenting adverse outcomes of TNF-inhibitors in CGD.

## Case Report

The patient is a young adult male, followed by our allergy and immunology service since early childhood, when he was initially diagnosed with X-linked CGD. He was born premature at 33 weeks of gestation and has no siblings. At 23 months of age, he developed persistent fever and was found to have right inguinal abscess and bilateral epididymo-orchitis due to *Serratia* species. A comprehensive workup for immune deficiency was undertaken. Dihydrorhodamine (DHR) assay demonstrated 0% of neutrophils with oxidation of DHR into rhodamine-123 following PMA activation. His mother's DHR assay showed two neutrophil populations, one with DHR oxidation and one displaying no oxidation following PMA activation, supporting a diagnosis of X-linked CGD. However, genetic testing results for his specific *CYBB* mutation are not available.

The patient was started on antibiotic and antifungal prophylaxis, along with interferon gamma-1b therapy at time of diagnosis. He remained infection free on this regimen between the ages of 2 and 14 years. Then at age 15 years, he developed severe *Nocardia* pneumonia. At this same age, he developed severe chronic abdominal pain associated with constipation and hematochezia. His initial colonoscopy was negative, and esophagogastroduodenoscopy (EGD) showed mild gastritis. *Helicobacter pylori* infection of the gastric antrum was identified. He was placed on triple therapy with amoxicillin, clarithromycin, and lansoprazole, but abdominal pain did not improve. Repeat colonoscopy 1 year later revealed granuloma formation in the duodenum and pseudopolyps in the jejunum, and a diagnosis of CGD-related colitis was made. He soon developed transsphinteric and intersphinteric anal fistulas that became repeatedly infected. Surgical debridement and treatment with prednisone were attempted to promote fistula healing and closure. However, perirectal abscesses would reoccur, with cultures intermittently positive for *Klebsiella pneumoniae* and MRSA. After 3 years of uncontrolled abdominal pain and poorly healing perianal fistula, patient was trialed on TNF-α inhibitor, infliximab, dosed at 5 mg/kg/dose.

**Figure 1:**
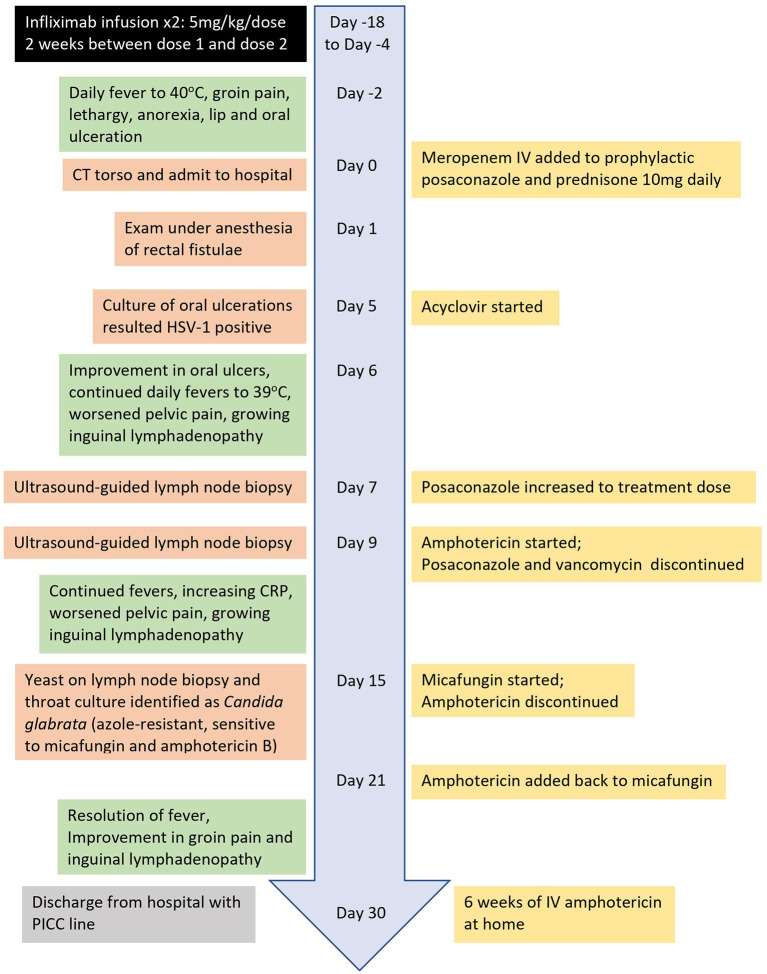
Timeline of hospitalization for *Candida glabrata* lymphadenitis.

**Figure 2:**
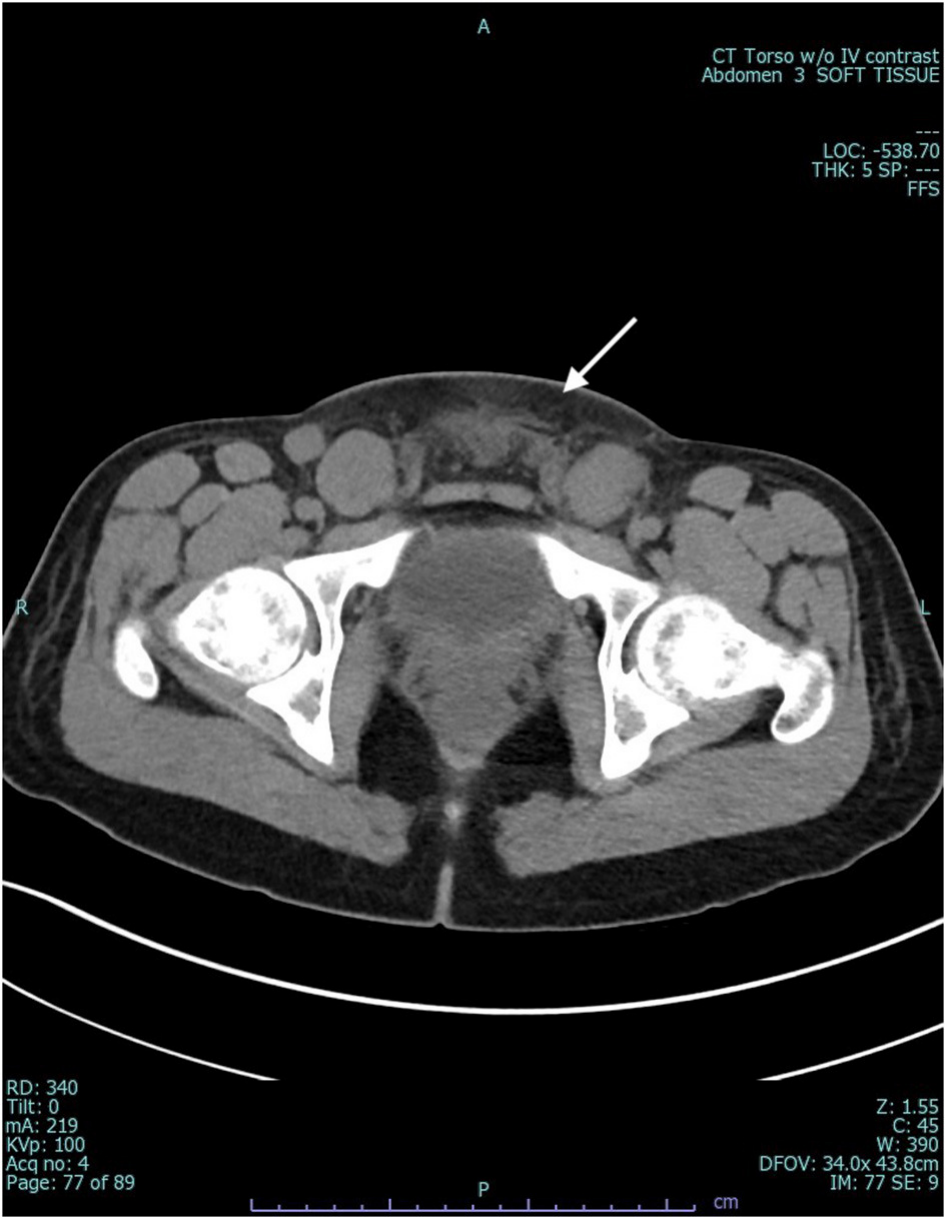
CT pelvis at admission demonstrating bilateral inguinal lymphadenopathy and subcutaneous fat stranding of lower abdominal wall.

Two days after second infusion of infliximab, given 2 weeks after dose 1, he developed fever of 40°C, associated with groin pain, new lip and oral ulceration, poor appetite, and lethargy. The patient was sent to the emergency department for further evaluation. He was placed on antibiotic therapy with meropenem and admitted to inpatient pediatrics service (Figure 1). His outpatient oral prednisone (10 mg daily) and prophylactic posaconazole were continued initially. CT of abdomen and pelvis revealed fatty stranding around pelvic lymph nodes and interval decrease in present transsphinteric and intersphinteric anal fistulas (Figure 2). The patient was taken to operating room for exam under anesthesia (EUA) to explore any potential deeper abscess formation. EUA was not suspicious for deep-seated visceral abscess formation. Oral ulcerations were cultured and found to be PCR positive for HSV-1, and the patient was placed on acyclovir.

Following admission, the patient spiked daily fevers averaging 39°C. While his oral ulcerations quickly improved after beginning a 10-day course of acyclovir, he experienced progressively increasing diffuse pelvic pain, and tender and growing inguinal lymphadenopathy, despite meropenem and acyclovir therapy. On Day 7 of admission, he had an ultrasound-guided core biopsy of left inguinal node, and antimicrobial coverage was expanded with addition of vancomycin and treatment doses of posaconazole. Histoplasma serology was weakly positive, but histoplasma antigen was negative. Blood cultures remained negative throughout admission. On Day 9, preliminary pathology results from lymph node biopsy revealed yeast. Vancomycin was discontinued and antifungal therapy was switched from posaconazole to amphotericin B. Daily fevers and painful lymphadenopathy continued, and CRP increased despite change in antifungal therapy. On Day 12 of admission, repeat MRI of abdomen and pelvis demonstrated increased pelvic lymphadenopathy.

On Day 15 of admission, yeast from lymph node biopsy speciated to *Candida glabrata* and was found to be azole resistant but sensitive to micafungin and amphotericin B. Yeast in throat cultures and from oral lesions also speciated to *Candida glabrata*. EGD showed no evidence of candidal infiltration into esophagus. Amphotericin was discontinued and micafungin was started. However, patient continued to be febrile. On Day 21, amphotericin B was added back to micafungin for synergistic effect. Patient clinically improved once started on amphotericin B and micafungin with resolution of fevers, improvement in groin pain and inguinal lymphadenopathy. After 30 days of admission, patient was discharged for an additional 6 weeks of intravenous amphotericin at home. Lymphadenitis fully resolved, and his perirectal fistulas greatly improved as well. Maintenance oral corticosteroids were discontinued and infliximab has not been reintroduced since the *Candida* infection. He has been maintained on conventional CGD prophylaxis of trimethoprim-sulfamethoxazole, itraconazole, and interferon-γ, with no further deep-seated infections in the 3 years since his infliximab course. More recently, he has now had a year-long history of recurrent superficial skin abscesses in the groin.

The patient is now exploring the treatment option of hematopoietic stem cell transplantation (HSCT) with his care team and has a matched unrelated donor identified.

## Discussion

Inflammatory bowel disease is a common complication of CGD, reported in approximately 1/3 to 1/2 of patients with CGD, and in an even higher proportion of patients with X-linked mutations (22, 23, 28). CGD-related colitis has similarities to Crohn's disease but with more rectal disease, fistulas, and more sharply defined granulomas (14). Oral corticosteroids, antibiotic therapy, and surgical options (fistulotomy, colectomy, etc.) are often used as initial treatment of CGD-related colitis, but treatment-refractory disease is common. While mortality is similar in patients with and without gastrointestinal manifestations, uncontrolled IBD significantly increases morbidity in patients with CGD (22). HSCT with matched sibling donors or matched unrelated donors has recently been shown to result in resolution of CGD-related colitis by 2 years following transplantation, but transplantation in CGD may be challenging and survival is best in young children (29). Thus, alternative treatment with the TNF-α inhibitor infliximab has been proposed to treat IBD in CGD when multiple medical and surgical options have failed, as was the case in our patient.

TNF-α is a pleiotropic cytokine of the innate immune system. It has been shown to be important in the immune response to many microbes including *Mycobacterium, Aspergillus, Histoplasma, Coccidioides, Toxoplasma, Cryptococcus*, and *Candida* species, mediating sequestration of pathogens within granulomas (25, 26).

In addition to impaired intracellular killing *via* oxidative burst, neutrophils in patients with CGD have been shown to have decreased expression of toll-like receptors-5 and receptors-9, decreased CD11b/CD18 expression associated with impaired phagocytosis, decreased CXCR1 expression associated with reduced neutrophil chemotaxis, and abnormal NET formation (6). However, patients with CGD have exuberant TNF-α production, possibly contributing to inflammatory complications such as colitis and aberrant granuloma formation (30). While blockade of TNF-α aims to reduce the hyperinflammation seen in CGD colitis, several case reports and series suggest it raises the risk of unchecked infection by common CGD pathogens.

Üzel et al. describe a series of five CGD patients with severe inflammatory bowel disease treated with the anti-TNF-α monoclonal antibody infliximab, following failure of conventional medical therapy with oral corticosteroids, NSAIDS (5-ASA), and immunosuppressive therapy (6-mercaptopurine), as well as one failure of adalimumab. Patients were started on 5 mg/kg dose of infliximab, with a second dose given 1 week later, and then subsequent doses given at 4-week intervals (27). Patients were on appropriate antimicrobial prophylaxis during the infliximab treatment, and three of five were receiving interferon-γ therapy. All participants of this series developed infections of varying degrees of severity during their infliximab treatment course, and there were two deaths.

Three of the five patients were male with X-linked CGD secondary to mutation in gp91^*phox*^. The two females carried autosomal recessive mutations in p47^*phox*^ and p22^*phox*^ respectively. Patient 1 (female, p47^*phox*^, 18 years old) developed a *Burkholderia cepacia* pneumonia following her 8th infliximab dose but was able to continue on therapy and achieve IBD remission and healing of her rectovaginal fistula after her 12th infusion of infliximab. Patient 2 (male, gp91^*phox*^, 25 years old) had significant healing of perianal fistulae by the 3rd infusion. However, after the 5th infusion, he developed an infected entrapped fistula track and associated *Candida lusitaniæ* inguinal lymphadenitis. Patient 3 (male, gp91^*phox*^, 16 years old) also had significant improvement of perirectal fistulas with infliximab with off and on dosing due to infections during therapy. He developed *Burkholderia multivorans* sepsis after the third dose, asymptomatic *Paecilomyces variotti* pneumonia after the fifth dose, and ecthyma gangrenosum following the 16th dose (wound cultures positive for *Pseudomonas aeruginosa, Stenotrophomonas maltophilia, Acinetobacter baumannii*, and *Candida glabrata*). Following dose 18, he developed fatal multiorganism infections including CMV colitis and viremia, and pneumonia with *P. aeruginosa, A. baumannii*, and *Aspergillus* hyphae identified on autopsy. Patient 4 (male, gp91^*phox*^, 16 years old) had colitis which failed to respond to adalimumab treatment prior to initiating infliximab. Infliximab achieved intermittent closure of fistula tracts with infliximab, resulting in trapped abscess pockets. After dose 5, he developed multiple hepatic microabscesses, which responded to treatment for presumed MRSA. Patient 5 (female, p22^*phox*^, 20 years old) had clinical response to infliximab, with resolution of rectovaginal fistulae and pelvic abscesses. However, following dose 12, she developed *Burkholderia cepacia* sepsis and subsequent fatal polymicrobial bacteremia with *Staphylococcus aureus, Candida krusei, Candida albicans*, and *Aspergillus terreus*.

Deffert et al. reviewed the literature to find 12 additional case reports of patients with CGD who received anti-TNF therapies. Only two of 12 cases reported infectious complications, and there were no deaths (31). One of the cases of infection was in a female with p47^*phox*^ mutation who developed *Aspergillus nidulans* in the brain and lungs following 4 years of therapy with infliximab. She had not been diagnosed with CGD until after her invasive fungal infection and therefore was not receiving antimicrobial prophylaxis (32). The second case with infectious complications was a 19-year-old male with X-linked CGD who developed a dental abscess, fungal pneumonia, and staphylococcal bacteremia following a single 5 mg/kg dose of infliximab for colitis (33).

A recent case series from France by Conrad et al. described 14 patients with CGD treated with at least one dose of anti-TNF therapy (infliximab or adalimumab) (34). Of the 12 patients in the series who received anti-TNF therapy for colitis, nine (75%) had rapid but incomplete improvement with the remaining three having no response. Of the 14 patients receiving anti-TNF therapy, seven developed infections (four with multiple infections) with a median time to first infection of 285 days from start of therapy (range 57–974 days). Infections included *Actinomyces* adenitis, cat-scratch disease, *Pseudomonas aeruginosa* folliculitis, *Salmonella* bacteremia, disseminated *Candida* infection, pulmonary mucormycosis, and several infections with unidentified pathogens (pneumonia, suppurative adenitis, and intra-abdominal abscess), but only two patients had to discontinue the TNF-inhibitor because of infections and there were no deaths. Ultimately, 10 patients underwent definitive treatment with HCST or gene therapy. The authors concluded that anti-TNF therapy should be considered as a temporary therapy for patients with severe, uncontrolled inflammatory complications of CGD, especially while awaiting HCST or gene therapy.

In the above reports, infectious complications after initiating infliximab or adalimumab were seen in a greater percentage of patients with X-linked CGD (10 of 16; 62.5%) than autosomal recessive CGD (4 of 15; 26.7%), suggesting that patients with X-linked CGD-IBD are at highest risk for infection with TNF-α inhibitor use (27, 31, 34). However, there were still some patients with X-linked CGD who tolerated extended courses of TNF-α inhibitors without serious infectious complication. It is possible that combined therapy with other immunosuppressants, such as the low dose prednisone our patient was receiving, may have contributed to risk of significant infection on TNF-α inhibitor therapy. Another consideration is that patient-specific factors within the X-linked CGD subgroup, including residual NADPH oxidase function, could result in better tolerance of TNF-inhibitors in some patients versus others. Our patient was free of serious infection for two years prior to his infliximab course and for three years after, suggesting that his brief infliximab course was the likely cause of his *Candida glabrata* infection.

In addition to TNF-inhibitors, several other biological therapies have been trialed in CGD-related colitis. Clinical response to the IL-1R antagonist anakinra has been reported in some patients with severe colitis (35), while others have seen no benefit with anakinra (36). No serious infectious complications have been reported in these trials of anakinra in CGD. A case report described significant improvement in treatment-refractory CGD colitis with the IL-23 antagonist ustekinumab, but the biologic had to be discontinued after severe pulmonary infection with an unidentified pathogen developed (37). Subsequently, a case series of nine patients treated with ustekinumab for CGD colitis showed clinical remission in four of nine patients, but there was lack of histologic remission in those undergoing colonoscopy (38). The anti-α4β7 integrin monoclonal antibody, vedolizumab blocks interaction of the α4β7 integrin with MAdCAM-1. It was reported to be effective in treating a patient with fistulating CGD colitis (39). Subsequently, a series of 11 patients with CGD colitis was reported in which seven patients achieved partial clinical improvement with vedolizumab, but none had long-term endoscopic improvement beyond six months of follow-up (40).

Colitis is a challenging complication of CGD. Many patients have complicated clinical courses, with poor disease control and/or significant side effects from therapies. While most trials of biologics to date for CGD-related colitis have led to either unsatisfactory results or serious infectious complications, it will be important to continue to investigate the use of new biological treatments as they become available, to determine if any of these agents can offer clinical benefit without increased infectious risks. While options for treating patients with CGD-related IBD prove to be limited, recent studies show the clear benefit of HSCT for CGD colitis, with good survival rates, especially in younger patients and those with a matched donor (29, 41). Curative therapy with HSCT is now being considered for an increasing number of patients with CGD, especially when options for treating disease complications such as CGD-related colitis remain limited.

## Conclusions

In our patient's case, we were confronted with the clinical dilemma of severe CGD colitis that was unresponsive to conventional therapies, prompting a trial of infliximab. Following the 2nd infusion of infliximab, patient developed HSV-1 stomatitis and azole-resistant *Candida glabrata* lymphadenitis, after being free of deep-seated infections for a 2-year period. TNF-inhibitor therapy has especially been associated with severe infections in X-linked CGD, though even some of these patients have been described that tolerate the therapy. Our patient's colitis has been improved in the last 3 years, yet given his history with infliximab and a lack of other biological options with well-described efficacy and safety in CGD colitis, proceeding with definitive therapy with HSCT is a key decision facing our patient and his care team.

## Data Availability Statement

The original contributions presented in the study are included in the article/supplementary material, further inquiries can be directed to the corresponding author/s.

## Ethics Statement

Ethical review and approval was not required for the study on human participants in accordance with the local legislation and institutional requirements. The patients/participants provided their written informed consent to participate in this study. Written informed consent was obtained from the individual(s) for the publication of any potentially identifiable images or data included in this article.

## Author Contributions

HL and RD contributed to the conceptualization, planning of the report, and contributed to the manuscript revision. RD wrote the first draft of the manuscript. All authors contributed to the article and approved the submitted version.

## Conflict of Interest

The authors declare that the research was conducted in the absence of any commercial or financial relationships that could be construed as a potential conflict of interest.

## Publisher's Note

All claims expressed in this article are solely those of the authors and do not necessarily represent those of their affiliated organizations, or those of the publisher, the editors and the reviewers. Any product that may be evaluated in this article, or claim that may be made by its manufacturer, is not guaranteed or endorsed by the publisher.
